# Transvenous lead extraction in conduction system pacing

**DOI:** 10.3389/fphys.2022.993604

**Published:** 2022-08-11

**Authors:** Nadeev Wijesuriya, Mark K Elliott, Vishal Mehta, Jonathan M Behar, Steven Niederer, Bruce L Wilkoff, Christopher A Rinaldi

**Affiliations:** ^1^ School of Biomedical Engineering and Imaging Sciences, King’s College London, London, United Kingdom; ^2^ Department of Cardiology, Guy’s and St Thomas’ NHS Foundation Trust, London, United Kingdom; ^3^ Cleveland Clinic Lerner College of Medicine of Case Western Reserve University, Cleveland, OH, United States; ^4^ Department of Cardiovascular Medicine, Deb Family Endowed Chair in Lead Management, Cleveland Clinic, Cleveland, OH, United States; ^5^ Heart, Vascular and Thoracic Institute, Cleveland Clinic London, London, United Kingdom

**Keywords:** CRT, conduction system pacing, transvenous lead extraction, his bundle pacing, left bundle area pacing

## Abstract

Conduction System Pacing (CSP) delivered by His Bundle Pacing (HBP) or Left Bundle Pacing (LBP) are exciting novel interventions in the field of Cardiac Resynchronization Therapy (CRT). As the evidence base for CSP grows, the volume of implants worldwide is projected to rise significantly in the coming years. As such, physicians will be confronted with increasingly prevalent and vital issues arising in long-term follow up, including the management of infected, malfunctioning, or redundant CSP leads. Transvenous lead extraction (TLE) is the first-line option for removal of pacing leads when indicated in these circumstances. The evidence base for TLE in the context of CSP is still in its infancy. In this article, we first provide a brief overview of TLE. We then examine the data on the long-term performance of HBP leads. Next, we describe the features of the Medtronic Select Secure 3,830 lead, and how experience of TLE of this lead in the paediatric population has informed our practice. Finally, we review the current evidence for TLE in HBP and LBP, and discuss how future studies can address gaps in our current knowledge.

## Introduction

**FIGURE 1:**
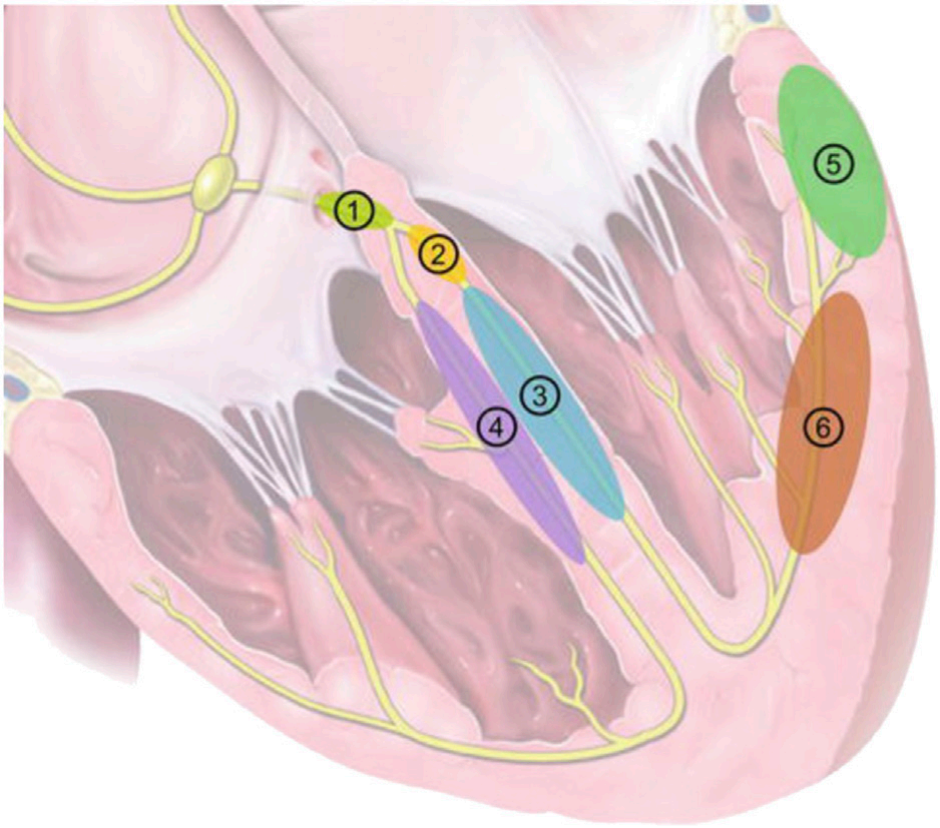
Pacing locations in CRT. 1: His-Bundle Pacing; 2: Left Bundle Branch Pacing; 3: LV septal pacing; 4: RV septal pacing; 5: Epicardial LV lateral wall pacing (conventional CRT); 6: LV endocardial pacing. Reproduced from reference 10, Karpenko et al., with permission.

The last decade has seen a steady expansion in the indications for Cardiac Implantable Electronic Devices (CIED) (Glikson et al., 2021). This has been driven significantly by the increasing evidence base for cardiac resynchronization therapy (CRT) in the treatment for not only its traditional indication of dyssynchronous heart failure (Cleland et al., 2005), but also for high grade atrioventricular (AV) block patients with mild-moderate left ventricular (LV) dysfunction (Curtis et al., 2013), and for patients undergoing AV node ablation for rate control of atrial fibrillation (AF) (Brignole et al., 2021). In recent years, conduction system pacing (CSP), delivered through His-Bundle pacing (HBP) (Vijayaraman et al., 2018) or Left Bundle pacing (LBP) (Zhang et al., 2019) have emerged as technologies which are not only viable alternatives to CRT, but potential first-line options in selected patients (Figure 1). (Arnold et al., 2018; Upadhyay et al., 2019; Karpenko et al., 2021; Žižek et al., 2021) Growing indications for CSP will create a growing patient populations requiring long term management of CSP systems.

The number of CIED implants now performed annually worldwide exceeds 1.2 million (Kusumoto et al., 2017), and there is a growing population of patients with transvenous devices requiring long-term management. It is well established that transvenous leads can fail or become infected at a rate of up to 2–4% (Wazni and Wilkoff, 2016; Rennert-May et al., 2020). The 2017 HRS consensus statement stipulates that infection is a class I indication for CIED extraction (Kusumoto et al., 2017). However, the guidelines for management of malfunctioning or redundant leads are less robust. There is growing evidence that the presence of multiple transvenous leads can increase risk of CIED infection (Hercé et al., 2013; Ann et al., 2015), venous thrombosis (van Rooden et al., 2004; Haghjoo et al., 2007; Bulur et al., 2010), tricuspid regurgitation (de Cock et al., 2000), inappropriate defibrillator shocks (Pfitzner and Trappe, 1998), and reduce the success rate of future extraction procedures (Segreti et al., 2014; Bongiorni et al., 2017a; Hussein et al., 2017). This increased risk has led experts to consider whether lead extraction may be superior to abandonment in the case of redundant or malfunctioning leads. This is reflected in the data from the European ELECTRa Registry (Bongiorni et al., 2017b), where 47% of extractions were performed for non-infective indications.

Initially, open heart surgery was a necessary procedure for the removal of intracardiac pacing leads, however over the last three decades, transvenous lead extraction (TLE) has become the first-line option for the majority of cases. This is an endovascular intervention which carries a lower morbidity compared to median sternotomy, particularly in a high-risk cardiac population. Despite advances in this field, TLE still carries an emergent complication rate of 1.8% and an in-hospital mortality rate of 1.2% (Perez et al., 2018), with the most serious complications being vascular laceration, cardiac avulsion, pericardial tamponade, haemothorax and thromboembolic events.

Since the advent of TLE, physicians have made complicated and nuanced decisions on both implanting and extracting devices based on balance of short-term risk versus long-term benefit. The widespread uptake of CSP presents a new challenge in this field. Current evidence on both the risk profiles and long-term outcomes of TLE are from transvenous pacing leads in the atrial, right ventricular (RV) or LV epicardial locations. Data on long-term performance and TLE of CSP systems, particularly LBP systems, is scarce thus far. However, it is likely that the rate of these procedures will increase significantly in the upcoming years, and as such, it is vital that the available technical and outcome knowledge is shared to better inform both physicians and patients.

This article first examines the current performance data for HBP leads, and the potential need for lead management procedures. We then review the data on TLE of the Medtronic SelectSecure 3,830 lead, the only lead with FDA approval for CSP, from the paediatric population, where it has been used for both atrial and RV pacing. Finally, we discuss TLE in HBP and LBP, both with regards to technical aspects of the procedure, and outcome data.

## Performance of HBP leads

Whilst studies suggest that HBP provides excellent electrical resynchronisation (Abdelrahman et al., 2018), implantation can be difficult, with success rates ranging from 56 to 95% (Bhatt et al., 2018; Vijayaraman et al., 2018; Zanon et al., 2019). Challenges have also emerged in longer term follow-up, such as ventricular under sensing and rising thresholds (Lustgarten et al., 2019). Vijayaraman reported in a non-randomised prospective study of 173 patients undergoing either RV pacing (RVP) or HBP for bradycardia that at 5 years follow up, the rate of lead revision was 6.7% in the HBP group versus 3% in the RVP group. In a retrospective study of 332 patients who underwent HBP, Abdelrahman et al. (2018) reported that 14% of patients experienced an increase in capture threshold of >2.5 mV over 2 years follow up. These findings were similar to those reported in a retrospective analysis of 274 patients performed by, where 24% of patients had an increase in HBP capture threshold of >2.5 mV, 17% of patients experienced loss of HBP capture, and 11% required lead revision for unacceptably high thresholds, at a mean follow-up of 22.8 months Teigeler et al. (2021) In the largest observational study of HBP reported that at a median follow-up of 3 years, 28% of patients had a pacing threshold of >2.5 mV. The median time to battery replacement was only 5.8 years, and 7.6% of patients required lead revision. Zanon et al. (2019) In keeping with these findings, the 2021 Medtronic Performance Report stated that at 5 years follow-up the survival rate of HBP leads was 91% compared to over 98% for the same lead model in the RV or atrial position (Medtronic, 2021).

Taken together, this available evidence suggests that despite excellent efficacy outcomes, the long term performance of HBP leads is in question. The rate of lead revision appears to be significantly greater than with RVP, even in the more contemporary studies, and this projects to an increase in the requirement for extraction of HBP leads moving forward.

## TLE of the Medtronic Select Secure 3,830 lead—Experience from the paediatric population

**FIGURE 2:**
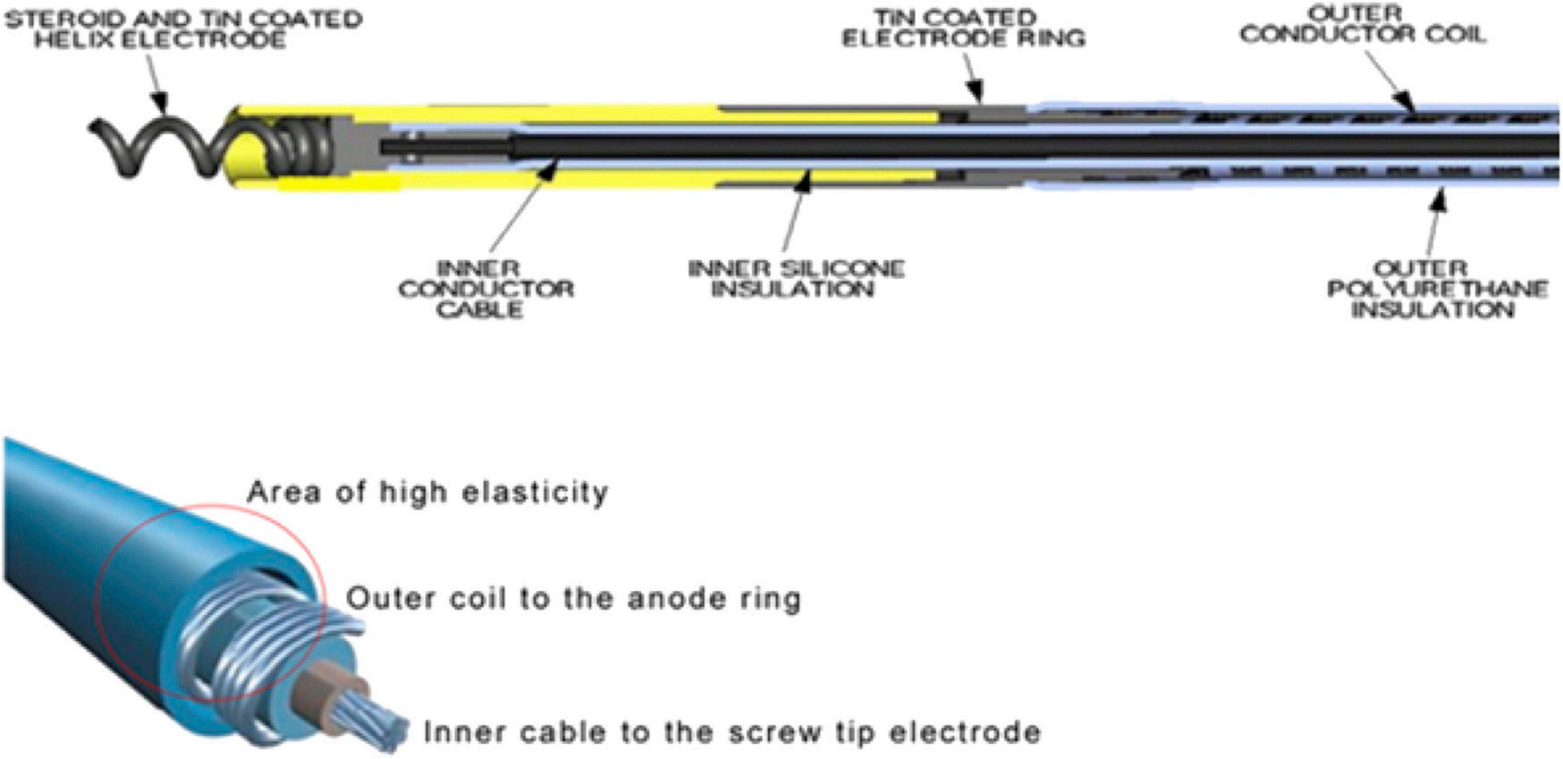
Design of the Medtronic 3,830 Lead. Reproduced from reference 31, Krainski et al., with permission.

The only lead currently with United States (United States) Food and Drug Administration (FDA) approval for HBP is the Medtronic Select Secure 3,830 (MDT 3830, Medtronic Inc, Minneapolis, MN). The MDT 3830 is a bipolar, narrow-body lead with a diameter of 4.1 French. An inner conductor cable for the tip electrode is covered with an inner silicone insulation. The outer ring conductor coil wraps around the silicone insulation and is itself covered by an outer polyurethane layer. The lead is lumenless and actively fixated with a nonretractable, exposed helix that is attached to a steroid-eluting tip (Figure 2). (Krainski et al., 2020) It is available in lengths of 59, 69, and 74 cm. It is implanted using either a steerable or pre-formed sheath depending on the site of delivery.

The MDT 3830 was initially developed for use in the paediatric population, where its small diameter gave it an advantage compared to standard leads. The advent of CSP has seen a significant increase in its use. Medtronic Performance reports state that there has been a 42% increase in United States registered implanters from 2016 to 2019, and the number of these leads implanted increased from 28,000 to 99,000 from 2016 to 2021 (Medtronic, 2021).

The lead has several features which make it distinct from standard pacing leads, namely, a lumenless design, a narrow calibre, and a cable-fixed exposed helix. These features impact upon the technical aspects of a TLE procedure. For example, the use of locking stylets is not possible, raising the need for extraction tools such as lead extenders and compression ties (Krainski et al., 2020). In addition, the high tensile strength 13lb (Medtronic, 2021) of the MDT 3830 due to the presence of an inner cable, and the presence of a non-retractable helix raises questions about the possible risks of myocardial avulsion when extracting leads with an increased dwell time.

Shepherd et al. (2015) performed a single-centre retrospective analysis of TLE in their paediatric and adult congenital heart disease (ACHD) population, comparing outcomes of MDT 3830 extractions with extractions of conventional leads. They found that 9/22 (41%) of MDT 3830 leads could be removed with manual traction alone, compared to only 2/35 (6%) of conventional leads. The remaining leads were all successfully removed with mechanical extraction tools. The authors noted that special consideration was needed when using a cutting sheath with the MDT 3830, as the sheath-lead size mismatch can lead to increased bleeding. Both univariate and multivariate analysis revealed that conventional leads were predictive of “complex procedure” defined as partial extraction or femoral extraction. It should be noted that in this study, the mean dwell time of the MDT 3830 was significantly lower than that of the conventional leads (4.1 ± 2.6 versus 6.7 ± 2.6 years, *p* = 0.01).

Garnreiter et al. (2015) also report a 6-year experience using the MDT 3830 in a paediatric/ACHD population. Over a mean follow up of 2 years, 11/198 leads required extraction (6%). Seven leads were >1 year old, and five of these were >2 years old. All leads were successfully removed by manual traction alone, with no intra-procedural complications.

## Extraction of his-bundle pacing and left-bundle pacing leads

Whilst CSP implants have become more prevalent than paediatric implants in recent years, data on TLE of leads in the HBP or LBP positions remains scarce. Evidence is limited to retrospective data sets and case reports.

Vijayaraman et al. (2019) performed a retrospective analysis of 30 patients who underwent TLE of HBP leads, with a mean dwell time of 25 ± 18 months 74% of extractions were due to unacceptably high thresholds. In 8 patients where the lead dwell time was <12 months, manual traction alone was successful in all cases. Of the remaining 22 patients, manual traction alone was successful in 18. The remaining four patients required mechanical extraction tools, and one extraction was unsuccessful. Reimplantation of a HBP lead was attempted in 17 patients, with the lead placed distal and superior to the original lead. HBP capture was achieved in 14 patients (86%). In 5 patients, LBP was attempted with a 100% success rate. This study demonstrates the feasibility and safety of HBP lead extraction and re-implantation to achieve capture of the His-Purkinje system.

Case reports have given us more detailed procedural insights into HBP lead extraction. Migliore et al. (2021a) report on the TLE of a 14 year old HBP system for infection. The Bulldog System (Cook Medical) and compression coil were used for fixation of the HBP lead. All leads were successfully extracted via the left subclavian vein using mechanical extraction tools (Evolution Shortie RL 11F, Evolution RL 13 F, and the Steady Sheath Evolution Tissue Stabilization Sheath 13 F) because of calcified adhesions in the subclavian and superior vena cava regions. The authors suggested that the high tensile strength of the MDT 3830 allowed successful extraction without the use of a locking stylet. They suggested that during such procedures, extraction of the conventional leads (e.g. atrial/RV pacing/ICD leads) should be performed first in order to improve adherence and thus avoid increased strain on the stylet-less lead.

Boczar et al. (2019) report on the removal of a HBP lead in a patient with ipsilateral venous occlusion. A 66 year old man with hypertrophic cardiomyopathy and permanent atrial fibrillation (AF) had a 1 year old system *in situ* which comprised of an ICD lead and a MDT 3830 lead which was deployed in the basal RV septum but was not achieving HBP capture. A revision to CRT plus HBP was planned. Contrast venography revealed left sided complete obstruction. Via a femoral approach, a Needle Eye Snare was introduced to the right atrium to stabilise the HBP lead. The lead was then extracted via the left subclavian vein with the aid of Byrd dilators (Cook Medical). The ICD lead was then successfully extracted, with an uneventful implantation of a new system.

With the increased interest in LBP, there have also been questions raised about TLE of these systems, given the deep septal location (see Figure 1) and potential for myocardial avulsion and iatrogenic ventricular septal defects. To date, there have been 3 case reports of LBP lead extraction, with all leads being <2 years old (Vijayaraman, 2020; Migliore et al., 2021b; Ponnusamy and Vijayaraman, 2021). In these 3 cases, uncomplicated extraction of these leads was performed by manual traction alone. It should be noted that, however, that there is concern about partial extraction in LBP systems. LBP involves boring the lead into the intraventricular septum, thus creating a fulcrum, and consequently a stress point at that fulcrum. When any structure is stressed it will break at its’ weakest point, metal fatigue at the fulcrum. This could lead to lead fractures, which have been documented (Chen et al., 2021), but may also be a source of partial extractions.

## Discussion

HBP and LBP are relatively novel procedures in the field of CRT. Since its advent, the volume of implants has increased steadily, and this is forecast to continue given the expansion in CRT indications and the widespread interest in CSP as not only an alternative to biventricular pacing, but perhaps a first-line option. Whilst there is a learning curve for this procedure, the available data suggests that the long-term system performance of HBP may be hampered due to issues with high thresholds (Medtronic, 2021). These issues may result in an increased requirement for extraction procedures moving forwards. Evidence in this area is limited to retrospective analysis of small cohorts, and what outcomes we can infer from larger cohorts in the field of paediatric/ACHD pacing. Feasibility of CSP extraction has been documented in these studies, and in particular, published case reports have provided valuable expert opinion in a niche field, where the lumenless and small calibre design of the MDT 3830 may require alternative extraction techniques, and considerations such as order of lead removal. The current data paints an optimistic picture of CSP TLE, with studies reporting good safety rates, and low use of mechanical extraction tools, however, care must be taken when interpreting this evidence base as it currently stands. In the first instance, retrospective data with small patient numbers may be subject to significant publication bias and under-reporting of complications. Secondly, it should be noted that in the majority of reported cases thus far, the lead dwell time is relatively low, and longer-term leads are likely to present significantly more challenges related to fibrosis and calcification. In addition, several technical questions remain in relation to the MDT 3830. Whilst suggestions have been made in case reports, formal testing has not yet been performed to determine the tensile strength of the MDT 3830 in the context of CSP, or the impact of differing lead preparation techniques. Finally, whilst the MDT 3830 is the most widely used lead for the delivery of CSP, there have been reports of LBP using stylet-driven leads such as the Solia S60 (Biotronik, SE & Co., KG) (de Pooter et al., 2021). There is as yet no published data on TLE of these systems, which may carry a different safety profile to the extraction of stylet-driven leads in conventional pacing locations.

These questions will need to be answered in order to facilitate the safe and effective management of long-term CSP leads in an ever increasing population of patients. It is important that prospective registry data on HBP and LBP extraction is gathered in order to determine short-term risks and long-term outcomes more reliably. Moving forwards, when considering CSP in the growing landscape of CRT, long-term lead performance and management will be as impactful as efficacy outcomes when making these nuanced and complicated decisions on behalf of our patients.
